# Modulating biofilm can potentiate activity of novel plastic-degrading enzymes

**DOI:** 10.1038/s41522-023-00440-1

**Published:** 2023-10-03

**Authors:** Sophie A. Howard, Ronan R. McCarthy

**Affiliations:** Division of Biosciences, Department of Life Sciences, College of Health and Life Sciences, Brunel University London, Uxbridge, UB8 3PH UK

**Keywords:** Applied microbiology, Biofilms

## Abstract

Plastic pollution is an increasing global issue desperately requiring a solution. Only 9% of all plastic waste has been recycled, and whilst recycling gives a second life to plastic, it is costly and there are limited downstream uses of recycled plastic, therefore an alternative is urgently needed. Biodegradation of plastic by microorganisms is a developing field of interest with the potential for bioreactors to be used alongside recycling to degrade plastic that may otherwise be sent to landfill. Here, we have identified two novel polyethylene terephthalate (PET) degrading enzymes through genomic mining and characterised their activity, including their ability to degrade PET. One of the main roadblocks facing the development of microbial enzymes as a plastic biodegradation solution, is that their efficiency is too low to facilitate development as bioremediation tools. In an innovative approach to tackle this roadblock, we hypothesised that enhancing a bacteria’s ability to attach to and form a biofilm on plastic could maximise the local concentration of the enzyme around the target substrate, therefore increasing the overall rate of plastic degradation. We found that increasing biofilm levels, by manipulating the levels of the second messenger, Cyclic-di-GMP, led to increased levels of polyester degradation in cells expressing novel and well characterised polyester-degrading enzymes. This indicates that modulating biofilm formation is a viable mechanism to fast track the development of bacterial plastic bioremediation solutions.

## Introduction

The accumulation of plastic waste is a major global issue threatening the environment, animals, and human health. Plastic waste production is rapidly increasing along with demand for plastic products, resulting in 12,000 million metric tonnes of plastic waste predicted to accumulate in the environment and landfill by 2050, despite increasing recycling trends^1–4^. Plastic waste in the environment results in animal entanglement and accidental plastic ingestion, leading to digestive blockages and choking hazards^5,6^. Plastic can cause physical damage to coral reefs, provide rafts for invasive species and pathogens, and carry pollutants and hazardous chemicals across oceans^7–9^. Plastic often breaks down into persistent microplastics in the environment, entering food chains and posing unknown long term affects to ecosystems and health^10,11^. Hundreds of hazardous chemicals are associated with plastic that can leach out of the plastic^12^. Specifically, leach from plastic waste has been shown to inhibit the growth of oxygen producing bacteria, disrupt soil microbial and invertebrate ecosystems, and endocrine signalling in marine mammals^13–15^. Globally, most collected plastic waste is taken to landfills, which risks leachate polluting the surrounding environment and endangering wildlife and humans in poorly managed sites^16^. Even in well managed landfill sites, leachate and greenhouse gases such as methane are by-products that need to be disposed of^17^. Alternatively, collected plastic waste is incinerated, whilst this can be used to generate electricity, there can be negative environmental consequences from toxic fumes and carbon emissions^18,19^. While recycling is an excellent method to enable plastic to have a second life, it is only part of the solution, as not all plastic types can be recycled and recycled plastic can often have fewer uses and limited reuses, so an alternative sustainable solution is urgently needed^20,21^.

The introduction of waste plastics into our ecosystems over the last 100 years has prompted scientists to investigate if microorganisms may have evolved the ability to degrade plastic waste. These investigations have led to the identification of a range of different bacterial and fungal enzymes that have been shown to degrade high prevalence environmental waste plastics^22–28^. An alternative solution to the plastic waste crisis could be the valorisation of waste plastic by plastic-degrading microorganisms into useful by-products, such as the common single use plastic polyethylene terephthalate (PET), which can be valorised into the bioplastic polyhydroxyalkanoate (PHA)^29^. Enzymes could either be purified and used directly or the bacteria that express the enzymes, whether native or heterologous, could be grown in batch culture to degrade plastic in bioreactors. However, a major roadblock that has arisen with many of the enzymes that have been identified so far is that their efficiency is too low to facilitate them being developed as viable plastic bioremediation tools. This is likely due to the presence of preferential carbon sources in their natural environment, so the enzymes do not need to be high efficiency for bacterial survival. A large research focus is now placed on improving enzyme efficiency so they can be more suitable for commercial application, predominantly through mutations in the active site to improve activity or mutations to improve enzyme thermostability. For example, single residue mutations in the substrate binding site of a cutinase, LCC, can improve activity, or additions of disulphide bridges can increase thermostability, resulting in an engineered PET depolymerase with nearly double the activity^30^. IsPETase, a PET hydrolase, has had considerable mutations studied, with many improvements made to its thermostability or activity^31–36^, most notably where machine learning was used to create a new version of this natural enzyme, FAST-PETase, with extensively improved activity^37^.

As an innovative way to tackle this roadblock, we hypothesised that enhancing bacteria’s ability to attach to and form a biofilm on plastic could increase the local concentration of the enzyme around the target substrate and maintain the enzymes in this location for longer by trapping them in the biofilm matrix, therefore increasing the overall rate of plastic degradation. Here, using genomic mining, we synthesised eight novel enzymes with the potential for polyester-degrading activity, two of which we confirmed were able to degrade PET. To assess if augmenting the levels of biofilm formation could improve their activity, we chose to modulate the levels of Cyclic-di-GMP (CdiGMP). CdiGMP is a eubacterial second messenger molecule that controls biofilm formation within almost all bacterial species, with high concentrations stimulating biofilm formation^38,39^. We introduced native and non-native diguanylate cyclases (DGC’s), which synthesise CdiGMP to our *E. coli* expression host to increase biofilm formation in the strains. We found that when biofilm was increased, polyester degradation was also increased for a well characterised polyester-degrading enzyme and for one of our novel enzymes. This provides a potential universal method to improve plastic degradation that could be used to fast track the development of real-world microbial plastic degradation solutions.

## Results

### Mining for novel polyester-degrading enzymes

Several known enzymes have previously been identified that can degrade plastics, namely, polyesters. Sequence similarities between these enzymes is quite high (Supplementary Fig. 1), lending itself to use the known sequences to search for potential novel polyester-degrading enzymes which may have improved activity profiles. Known PET-degrading enzymes were collated (Supplementary Table 1) and used to identify novel proteins with potential polyester-degrading activity. Through homology searches to the known enzymes, novel homologues with ~50–80% similarity were identified. A process of subtractive reduction was used to select 8 enzymes that were distinct from known enzymes but that we predicted may have activity based on sequence conservation and tertiary structure homology (Table 1). All 8 enzymes possess the known PET-degrading catalytic triad (Ser160, Asp206 and His237 – residue numbers for IsPETase) and conserved Gly–x1–Ser–x2–Gly motif (Fig. S1)^40^.

**Table 1:** Potential novel polyester-degrading enzymes selected through genomic mining.

New enzyme	Enzyme description	Species	ID	Most similar known PET-degrading enzymes		
				Known enzyme name and accession	Genomic similarity^a^	Structural similarity^b^
Dh1	Dienelactone hydrolase family protein	*Lentzea fradiae*	WP_090047116.1	Tcur1278 (ACY96861)	59.2%	RMSD 1.847 over 264 residues
Dh2	Dienelactone hydrolase family protein	*Marinobacter nanhaiticus*	WP_085988667.1	PET2 (ACC95208)	65.1%	RMSD 1.894 over 272 residues
Dh3	Dienelactone hydrolase family protein	*Alkalilimnicola ehrlichii*	WP_116302305.1	PET2 (ACC95208)	67.9%	RMSD 0.804 over 272 residues
Dh4	Dienelactone hydrolase family protein	*Cytophagales bacterium*	MBC7956640.1	IsPETase (GAP38373)	79.1%	RMSD 0.213 over 256 residues
Dh5	Dienelactone hydrolase family protein	*Aquabacterium sp*	MBI3384080.1	PET12 (AKJ29164)	54.1%	RMSD 2.291 over 272 residues
Dh6	Dienelactone hydrolase family protein	*Caldimonas taiwanensis*	WP_062195544.1	IsPETase (GAP38373)	49.2%	RMSD 1.284 over 256 residues
Dh7	Dienelactone hydrolase family protein	*Caldimonas manganoxidans*	WP_019560450.1	IsPETase (GAP38373)	49.2%	RMSD 1.446 over 256 residues
C_1	Cutinase	*Saccharothrix carnea*	PSL56014.1	Tcur1278 (ACY96861)	62.4%	RMSD 3.264 over 272 residues

^a^Genomic similarity information is the percent identity from a global sequence alignment using EMBOSS Needle between the novel enzyme and its closest known PET-degrading enzyme.

^b^Structural similarity was calculated using the ‘cealign’ command in PyMOL of the Phyre2 predicted structures of the novel enzymes, Tcur1278, PET2 and PET12 and solved structure of IsPETase (PDB 5XJH).

**Fig. 1:**
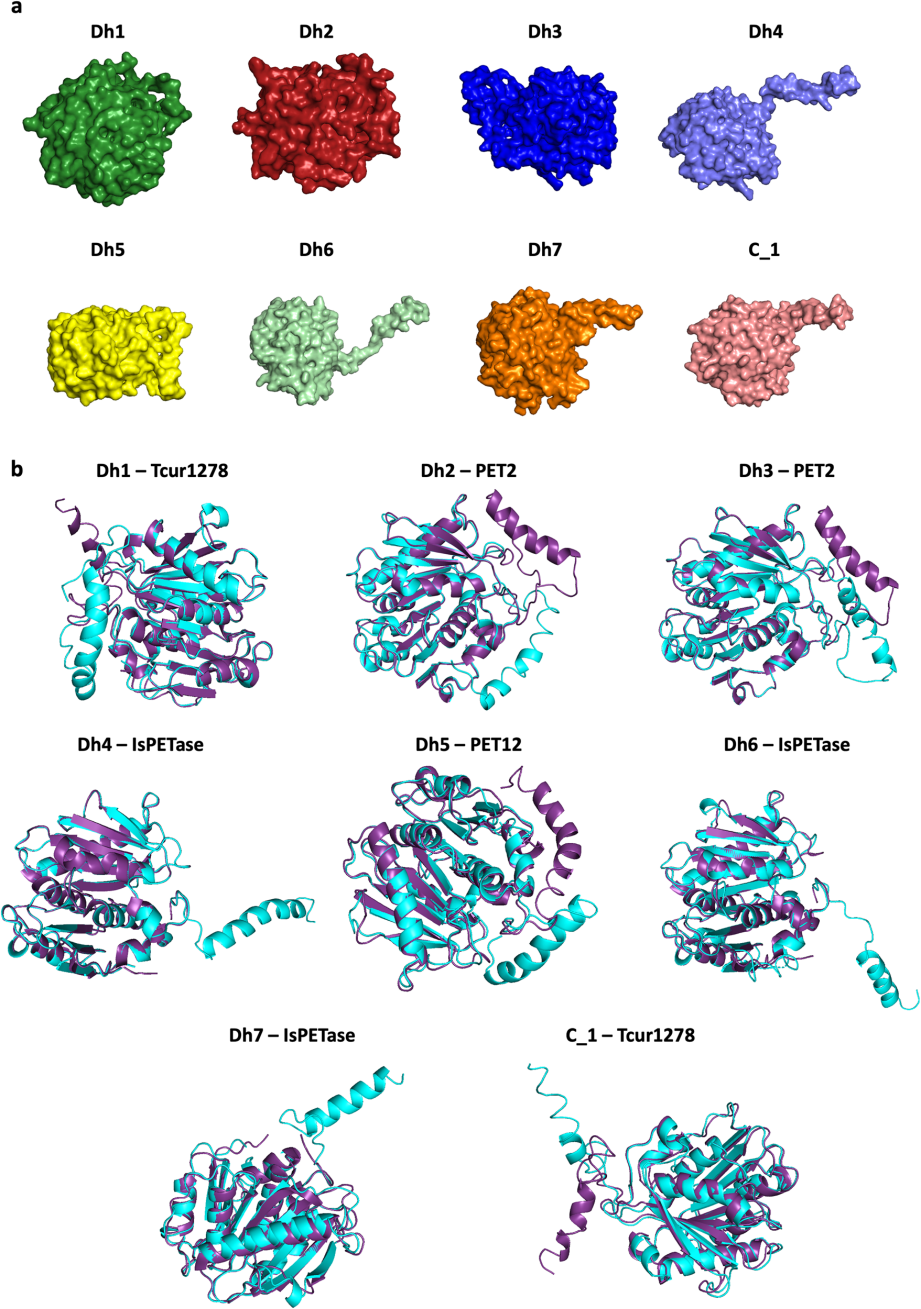
Modelling of selected novel enzymes and alignment with known PET-degrading enzymes. **a** Predicted structures of novel enzymes selected for synthesis with surface structure displayed. **b** Structural alignment with closest known PET-degrading enzyme, novel predicted enzyme in cyan, known PET degrader in purple; IsPETase (PDB 5XJH), Tcur1278, PET2 and PET12 were Phyre2 predicted structures with 90%, 86% and 90% of residues modelled at >90% confidence, respectively.

**Fig. 2:**
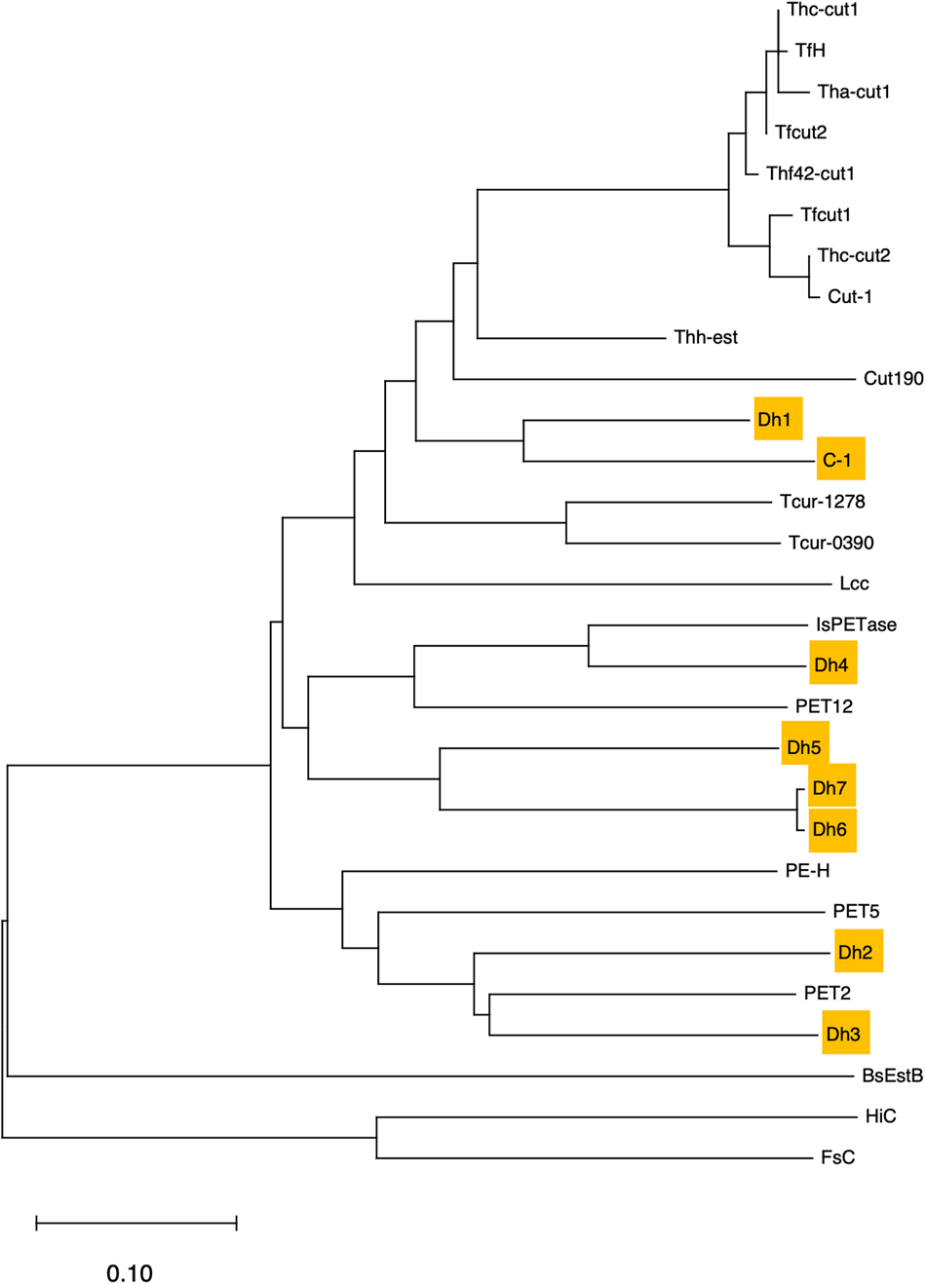
Phylogenetic analysis of potential novel polyester-degrading enzymes. A neighbour-joining tree was constructed using the *p*-distance of the enzyme protein sequences. Potential novel polyester-degrading enzymes highlighted.

The sequences of Dh1 and C_1 classify them as type I PET-degrading enzymes, Dh2 and Dh3 as type IIa, Dh4 as type IIb, and part of the subsite II of Dh5, Dh6 and Dh7 matches type I but they have an extended loop region which is only found in type II enzymes. The predicted structures of these enzymes were generally high confidence, the only low confidence area was a small region at the N-terminus (Fig. 1), which is usually less conserved between PET-degrading enzymes (Fig. S1)^40^. Of the residues that were modelled with >90% confidence, Dh1 had 86%, Dh2 had 89%, Dh3 had 87%, Dh4 had 91%, Dh5 had 91%, Dh6 had 92%, Dh7 had 92%, C_1 had 90%. Dh4, Dh6, Dh7 and C_1 all present with a predicted protrusion, this is at the N-terminus where there is low confidence. Alignment of the predicted structures with the closest known PET-degrading enzyme showed that the majority had a root mean square deviation (RMSD) below 2, which is considered a good alignment (Table 1). Phylogenetic analysis of the known and novel enzymes shows that we selected a variety of novel enzymes across different clades and enzyme types, confirming the diversity among our panel of candidate enzymes (Fig. 2).

### Screening of potential novel polyester-degrading enzymes for activity

**Fig. 3:**
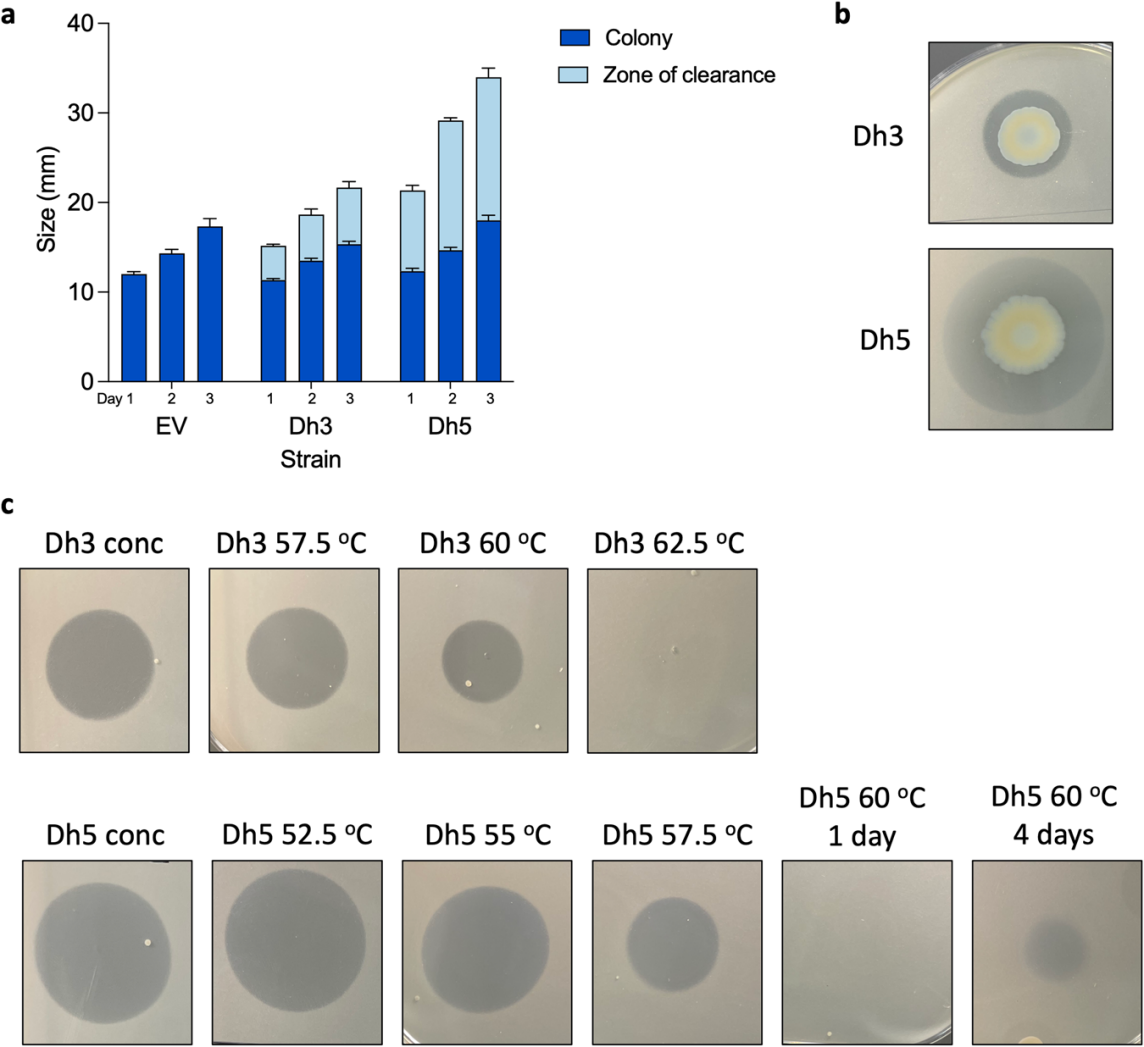
PCL degradation and thermostability of novel enzymes. **a** PCL clear zone assay of novel polyester-degrading enzymes. Dh3 and Dh5 are expressed in BL21 (DE3) with zone measurements on day 1, 2 and 3, mean and standard error of the mean (SEM) of 3 biological repeats, empty vector (EV) was used as a control for no PCL clearance. **b** PCL clear zone images, photographed on day 3, representative image. **c** Thermostability of secreted Dh3 and Dh5 was assessed by heat treating for 20 minutes and spotting on 1% PCL LB agar plates and incubation at 37 °C. Top row, Dh3 untreated and concentrated, heat-treated at 57.5 °C, 60 °C and 62.5 °C after 24 h. Bottom row, Dh5 untreated and concentrated, heat-treated at 52.5 °C, 55 °C, 57.5 °C, 60 °C after 24 h then 60 °C after 4 days. Representative images of three biological repeats.

The novel selected enzymes were codon optimised for expression in *E. coli*, cloned into pET20b and transformed into *E. coli* BL21 (DE3)^41^. For the 8 novel enzymes synthesised, polyester-degrading capacity was screened using polycaprolactone (PCL) clear zone assays. PCL is a model substrate for degradation of certain plastics because it can be easily dissolved into agar and as a polyester, it can be used to screen for potential degradation activity against polyester-based plastics, such as PET. Large zones of PCL degradation were seen for only two enzymes, Dh3 and Dh5 (Fig. 3a, b). Both enzymes are characterised as dienelactone hydrolase family proteins. Dh3 (WP_116302305.1) is from *Alkalilimnicola ehrlichii*, this enzyme is 67.9% identical to known PET-degrading enzyme PET2. *A. ehrlichii* is a Gram-negative arsenite-oxidising haloalkaliphilic gammaproteobacteria. Dh5 (MBI3384080.1) is from *Aquabacterium* sp., whilst the exact species is unknown, it was identified in a groundwater metagenome analysis. Dh5 is 54.1% identical to known PET-degrading enzyme PET12. The genes for both Dh3 and Dh5 already contain a signal peptide, so they will also likely be secreted.

The supernatant of strains expressing either Dh3 or Dh5 was tested and shown to have PCL degrading activity, confirming that both enzymes are indeed secreted (Fig. 3c). The thermostability of these two novel enzymes was also assessed by collection of the supernatant from induced cultures and heat treating them for 20 minutes before spotting on PCL containing agar plates (Fig. 3c). Dh3 is stable at 57.5 °C but then loses some of its activity at 60 °C, with all activity lost at 62.5 °C. Dh5 has a minor loss in activity at 55 °C, more so at 57.5 °C then substantial loss at 60 °C, with a clear zone only appearing after a few days. These properties indicate that we have identified two novel PCL-degrading enzymes that are secreted, relatively thermostable, and based on their homology, both genomically and structurally to known PET degraders, are likely to have high potential to also degrade more recalcitrant polyesters.

### PET-degrading activity of novel enzymes

**Fig. 4:**
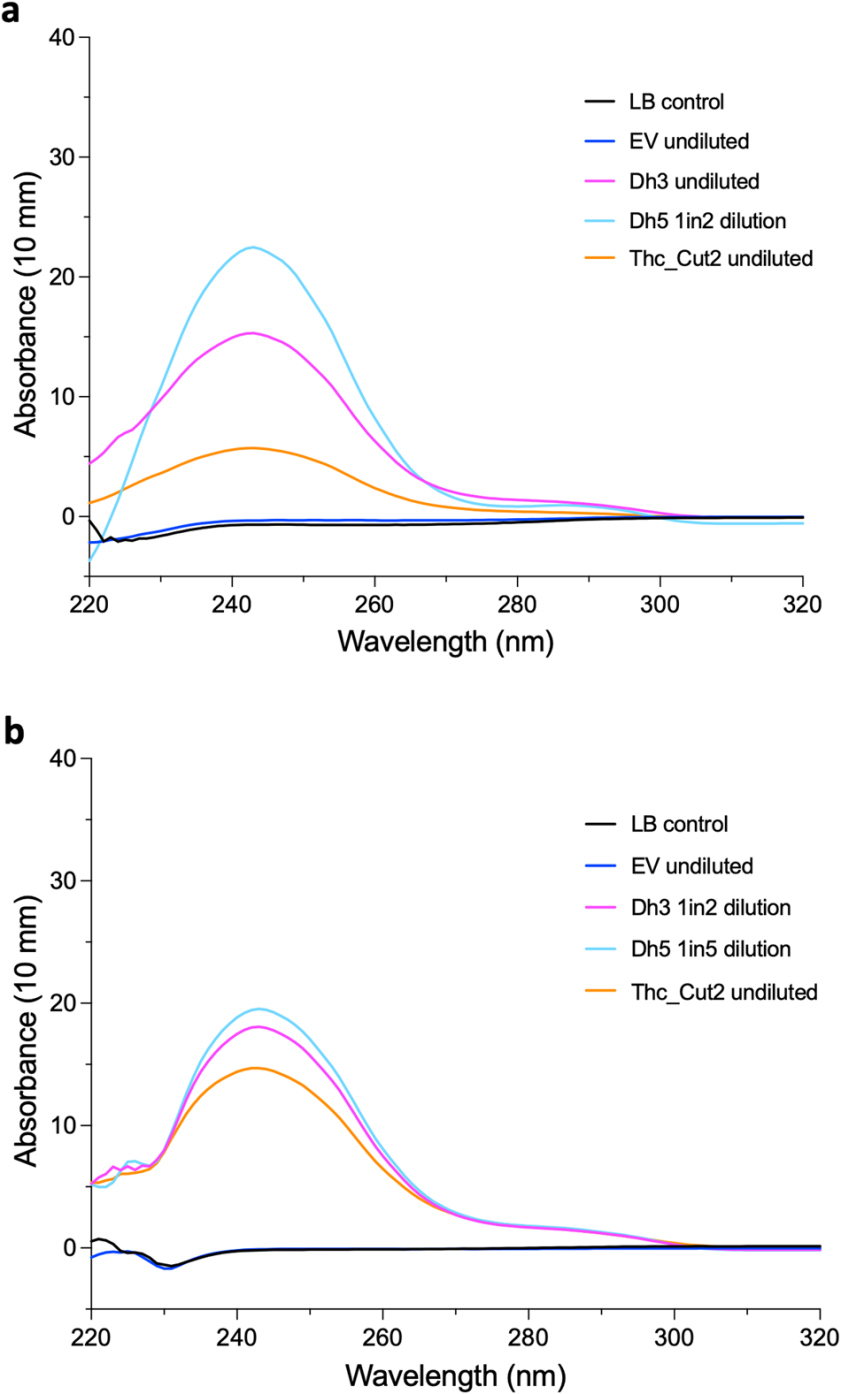
PET degradation by novel enzymes. Nanodrop absorbance readings of PET powder mixed with supernatant containing polyester-degrading enzymes. Absorbance readings trimmed to 220-320 nm. **a** Supernatant mixed with PET powder for 5 days. Undiluted Dh5 resulted in a peak above the detection limit so was diluted in distilled water by 1 in 2. **b** Acetone precipitated proteins dissolved in enzyme reaction buffer mixed with PET powder for 5 days. Undiluted Dh3 and Dh5 resulted in a peak above the detection limit so were diluted in reaction buffer by 1 in 2 and 1 in 5, respectively and these diluted values plotted instead. Mean curves of 4 biological replicates from the same day.

Whilst we have confirmed that these enzymes are capable of degrading PCL, we wanted to confirm activity on significantly more recalcitrant plastic such as PET. We selected a known PET-degrading enzyme, Thc_Cut2 (GenBank ADV92527), as a positive control for inclusion in PET degradation assays^22^. Thc_Cut2 was cloned into pET20b and expressed in BL21 (DE3) as well, and a nanodrop-based absorbance assay was used to detect the presence of PET breakdown products terephthalic acid (TPA) and Mono-(2-hydroxyethyl)terephthalic acid (MHET), which form a peak between 230 nm and 260 nm^40,42,43^. Firstly, different concentrations of TPA were measured on the nanodrop to confirm the peak presence from this substrate and change in peak height as concentration changed (Supplementary Fig. 2). Supernatant from induced BL21 (DE3) cultures expressing known and novel polyester-degrading enzymes were normalised for total protein concentration and mixed with PET powder. Supernatant containing Thc-Cut2 mixed with PET powder displayed a peak between 230 nm and 260 nm (Fig. 4a), confirming degradation of PET by this enzyme and validating the assay. We then tested Dh3 and Dh5 to assess their ability to degrade PET. Supernatants containing either of the novel enzymes mixed with PET powder displayed a high peak between 230 nm and 260 nm, showing substantial PET degradation (Fig. 4a). Dh5 was above the limit of detection so was diluted 1 in 2 (Supplementary Fig. 3a).

To further validate these results, we precipitated the secreted proteins to remove other molecules from the supernatant that may be limiting enzyme activity and resuspended the proteins in an optimised buffer that may promote stronger activity, which had previously been used by the assay developers for IsPETase^42^. To confirm that acetone precipitation did not denature the enzymes, the resuspended enzyme pellet was spotted on 1% PCL LBA plates and clear zones indicating activity were observed (Supplementary Fig. 4). Precipitated supernatant containing Thc_Cut2 had the best activity against PCL with the largest and clearest zone, followed by Dh5, then Dh3 had a smaller and less clear zone on PCL. The precipitated protein concentrations were normalised and then used in the nanodrop PET degradation assay. The absorbance peaks of the precipitated proteins were higher than for the supernatant (Fig. 4b), indicating greater activity in these conditions, which is especially key given that total protein concentration was half in the precipitated samples, yet activity was approximately double. Again, Dh5 had to be diluted, this time 1 in 5, and Dh3 had to be diluted 1 in 2 since undiluted samples were above the limit of detection (Supplementary Fig. 3b, c), indicating as expected that that they work more efficiently when in the reaction buffer compared to LB. We have identified two novel PET-degrading enzymes that can convert PET to its breakdown products efficiently and may hold significant bioremediation potential. Additionally, although PCL-degrading activity was not observed, the other novel enzymes synthesised were checked for PET-degrading activity using this assay. Supernatant samples and precipitated samples of the other novel enzymes, Dh1, Dh2, Dh4, Dh6, Dh7 or C_1 did not show any peak for PET degradation products (Supplementary Fig. 5).

### Modulating biofilm to improve plastic-degrading activity

**Fig. 5:**
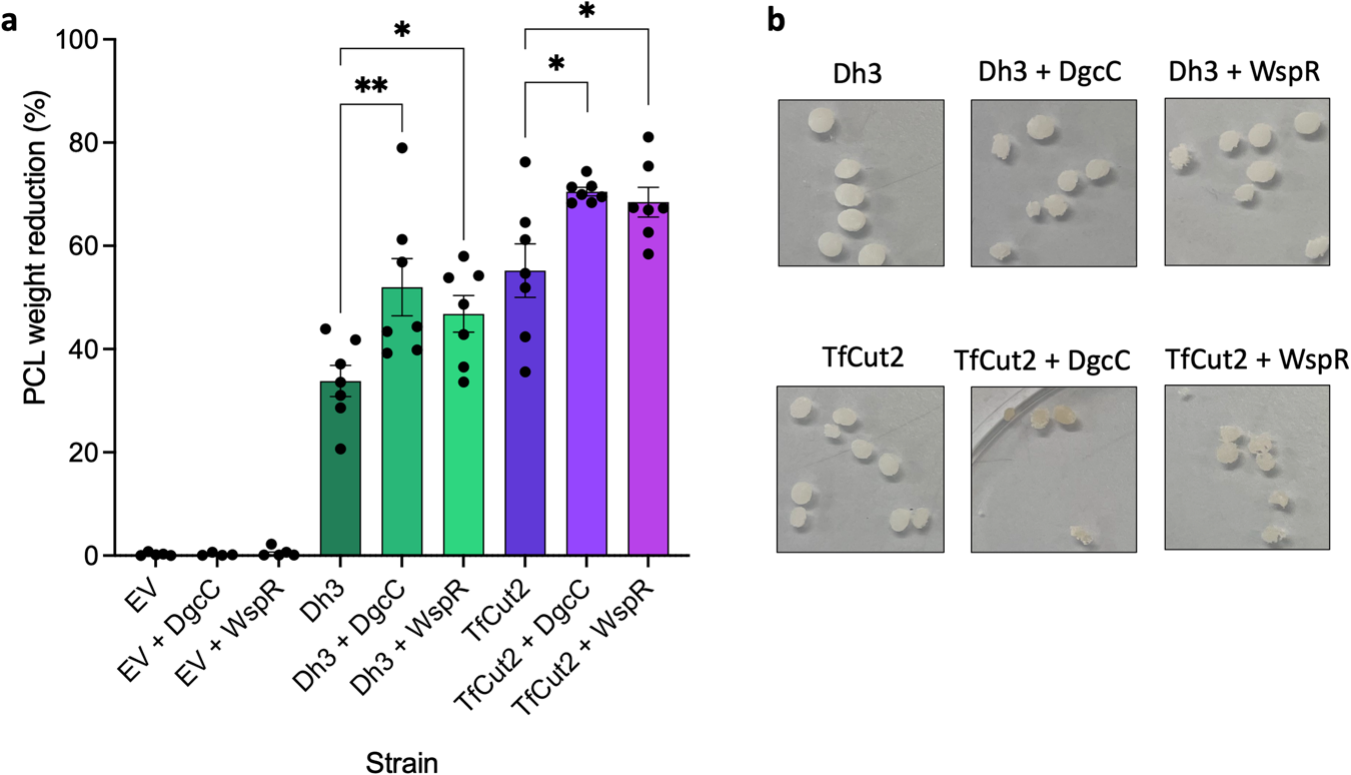
PCL degradation by polyester-degrading enzymes with increased biofilm formation. **a** PCL weight loss after 5 days when incubated with BL21 (DE3) empty vector, Dh3 or TfCut2 with and without co-expression of DgcC or WspR. Mean and SEM of seven independent experiments, ordinary one-way ANOVA with multiple comparisons was used to compare whether addition of DGC significantly changed PCL weight loss to the strain without. *p*-values are 0.0051 for Dh3 vs Dh3 + DgcC and 0.0356 for Dh3 vs Dh3 + WspR, F 7.511. *p* values 0.0116 for TfCut2 vs TfCut2 + DgcC and 0.0133 for TfCut2 vs TfCut2 + WspR, F 1.429. **b** Examples of PCL beads from one experiment for Dh3 (top) and TfCut2 (bottom) with and without DgcC and WspR.

Whilst these novel PET-degrading enzymes displayed good PET degradation activity compared to a well characterised PET-degrading enzyme, it is likely that their efficiency is still below what would be required for real world plastic bioremediation applications, as is the case with all native PET-degrading enzymes identified to date. To overcome this issue, rather than modifying the enzymes, we wanted to take an innovative approach and modify the behaviour of the bacterial host to maximise enzyme activity, specifically by increasing biofilm formation. By using biofilm as a modification, we hypothesised that increased biofilm formation in the culture could enhance the rate of plastic degradation. The principal benefit of this approach is that it will bring the bacteria in closer proximity with waste plastic, which in turn will increase the local concentration of the degrading enzymes around their target substrate. The other major benefit is that the polysaccharide matrix will limit the enzymes from being washed off the plastic. To address this hypothesis, we added a second plasmid to the *E. coli* BL21 expression system that encoded for DgcC (an *E. coli* diguanylate cyclase) or WspR (a *Pseudomonas aeruginosa* diguanylate cyclase), both previously related to increased biofilm formation^44,45^. To confirm that in this background both diguanylate cyclases were capable of inducing biofilm formation, a biofilm assay was performed and colony forming units (CFU) on PCL bead surface was assessed. The biofilm assay confirmed that both expression of DgcC and WspR increased biofilm formation as observed through the purple crystal violet ring at the air-liquid interface of the culture (Supplementary Fig. 6a). The CFU assay confirmed that significantly more bacteria attach to the surface of PCL when DgcC or WspR are expressed (Supplementary Fig. 6b). To test whether increasing biofilm formation would lead to increased levels of plastic degradation, the novel PET-degrading enzyme Dh3 and a known PET-degrading enzyme TfCut2 (GenBank: CBY05530)^22,46^ were tested. The enzymes were co-expressed with DgcC or WspR and the effect on PCL weight loss was measured. Weight loss was used rather than clear zone assays because it would allow for biofilm formation in liquid culture which is more representative of a potential bioreactor application. The strains with increased biofilm formation (expressing a DGC) also displayed increased PCL weight loss after 5 days incubation (Fig. 5a). Representative images of the degraded PCL beads after 5 days are included to show the difference in PCL degradation between the enzymes with normal levels of biofilm and the enzymes with increased biofilm (Fig. 5b). This suggests that batch culture plastic degradation systems could therefore improve their efficiency by co-expression of DGC’s to create high-biofilm forming expression systems.

## Discussion

The increasing plastic waste and pollution problem is a major environmental threat and current mitigation initiatives such as recycling are not having the impact required to tackle this threat. Here, we identified novel PET-degrading enzymes found through genomic mining. We synthesised and tested 8 proteins and demonstrated that 2 had excellent activity on PCL and PET. Dh3 and Dh5 are ~55–70% identical to known PET-degrading enzymes, showing how distinct the enzymes can be, they also only share 46% identity to each other, presenting themselves as novel PET-degrading enzymes. Interestingly, the structural alignment of Dh5 and its closest known PET-degrading enzyme, PET12, had one of the highest RMSD of 2.291, but the protein still maintained function, compared to the structural alignment of Dh4 and IsPETase which had a RMSD of 0.213, yet Dh4 showed no activity on PCL or PET powder in our experimental conditions. However, it is noted that expression, folding, and function were not confirmed for the enzymes, and some may be functional in their native host when potential required co-factors, post-translational modifications or support enzymes may be present. Additionally, longer time frames or different PET substrates may have yielded a result. Therefore, we cannot definitively say that the other novel enzymes found through genomic mining do not degrade plastic. Additionally, wider search parameters, such as enzymes with > 80% or < 50% identity, or screening using additional known PET-degrading enzymes may have yielded more novel PET-degrading enzymes, which can be assessed in future studies.

We used PCL degradation as a method to screen the synthesised enzymes, then confirmed the activity on PET by using detection of the breakdown products, TPA and MHET. To detect the presence of PET breakdown products, we used a recently described absorbance based methodology^42^. Unlike the original methodology which uses purified proteins, we were able to take the supernatant from induced cultures and mix this directly with PET powder, as well as extract the supernatant proteins through acetone precipitation to perform the reaction in enzyme reaction buffer, which greatly improved the enzyme activity and demonstrates the versatility of this assay. Both methods do not require the enzyme to be purified, just secreted, and it is also likely that non-secreted proteins could be used in this method as well, if obtained in supernatant form through cell lysis. It is noted though that not all enzymes would resist acetone precipitation, so confirmation of activity would be required after precipitation, which we did by spotting the enzyme mixture on PCL plates to detect clear zones.

Whilst our BL21 (DE3) expressing Thc_Cut2 degraded PCL better than Dh3 and Dh5, both of our novel PET-degrading enzymes degraded PET powder to a higher amount than Thc_Cut2 under our experimental conditions. PET-degrading enzymes can be classified into 3 types, type I, IIa and IIb, the catalytic triad and subsite I are conserved among all PET-degrading enzymes^40^. Thc-Cut2, the known enzyme used in our PET breakdown detection is a type I enzyme. Dh3 classifies as type IIa, although the first residue of subsite II is methionine whereas it is normally threonine, valine or leucine. The difference in type could contribute to the observed increased activity for Dh3 compared to Thc_Cut2. Interestingly, Dh5 does not fit with the typical classifications and gave much higher efficiency for PET degradation in these conditions. The first three residues of Dh5 subsite II match with type I (T, A, H), however the last two residues do not classify with the three types or match to other enzymes; a leucine is present at the 4^th^ position, whereas all other known enzymes possess phenylalanine, tyrosine, serine or threonine, and the 5^th^ position is a methionine, also shared with Dh6 and Dh7, but not by known enzymes which possess an asparagine or glutamine. Dh5, Dh6 and Dh7 also all have the extended loop seen only for type IIa/b enzymes. These differences in Dh5 could contribute to its observed higher activity, however, comparisons to more enzymes of all types and point mutations would have to be made to confirm why Dh5 appears more potent than Thc_Cut2 and Dh3, as well as purifying the enzymes to compare them directly.

We chose to take an innovative approach to improve enzyme efficiency by modifying the bacterial behaviour and increase biofilm formation as this has not been investigated previously and could be applied more universally than single residue mutations. Although the enzymes are secreted, we hypothesised that the concentration of enzyme around the plastic would likely be higher if the bacteria were to form a biofilm matrix around the plastic. The biofilm would also reduce the amount of enzyme washed away in the culture. Biofilm formation is common in the environment, including on waste plastics^47–49^. Natural biofilm formation on plastic has been linked with good plastic degradation previously, suggesting this may be a behaviour that has also evolved in marine microbial ecosystems contaminated with plastic^49–51^. We found that when either a native *E. coli* DGC or a *P. aeruginosa* DGC were co-expressed with the enzymes, creating high biofilm forming strains, the weight loss of PCL was increased for both the novel enzyme Dh3 and a known enzyme TfCut2, providing a proof of principle of the concept. As the enzyme degrades PCL in the same way it degrades PET, it stands to reason that degradation of PET would also be improved in high biofilm forming strains. This should be applicable to many enzymes to help them reach higher efficiency and could be used in batch culture reactors that degrade plastic, providing an innovative method to improve plastic-degrading enzyme efficiency in future.

## Methods

### Genomic mining

Known PET-degrading enzymes were collated and their protein sequences were used to search for novel potentially polyester-degrading enzymes using NCBI Protein Blast. BLAST hits were selected if their identity was between ~50 and 80% to ensure they were distinct proteins but closely related enough that function could be maintained.

### Phylogenetic trees and predicted structures

Enzyme protein sequences were aligned and input into phylogenetic trees using MEGA (version 11.0.11), alignment was performed using the MUSCLE algorithm, distance was measured using pair-wise *p*-distance with standard settings, a neighbour-joining tree was constructed using the *p*-distance with standard settings. Predicted protein structures were generated with Phyre2^52^ and uploaded into PyMOL for visualisation and alignment, using command ‘cealign’.

### Generation of constructs and bacterial growth

GenScript was used to synthesise the enzyme genes; potential polyester-degrading enzymes were cloned into pET20b and DGC’s were cloned into pCOLADuet-1. Sequences were codon optimised for expression in *E. coli*. Each novel enzyme sequence contained a predicted Sec signal peptide so were cloned using NdeI to replace the vector signal peptide with the enzyme native signal peptide. *E. coli* BL21 (DE3) was used throughout with Ampicillin 100 μg/ mL (for pET20b) and Kanamycin 50 μg/ mL (for pCOLADuet-1) used for plasmid selection and maintenance. All strains were grown at 37 °C static (plate) or with agitation at 180 rpm (flasks). For plasmid expression, cells were either plated on agar containing 500 μM Isopropyl β-D-1-thiogalactopyranoside (IPTG) for induction or 1 mM IPTG was added to cultures once they reached OD_600_ ~ 0.6.

### PCL clear zone assay

PCL (Sigma-Aldrich, Mn 80,000) was dissolved in acetone with agitation at 50 °C then mixed with LB agar solution (distilled water, 1.5% w/v agar-agar (Fisher), 2% w/v LB medium (Fisher)) to a concentration of 1% w/v PCL after acetone evaporation^27^. After autoclaving, the agar was cooled slightly (but not solidified to keep PCL in solution), Ampicillin, Kanamycin and IPTG were added where appropriate and poured into plates, resulting in cloudy agar plates. Overnight cultures of bacteria were grown in LB and OD corrected to OD_600_ 3, 20 uL of this was spotted onto the 1% PCL LBA plates and grown at 37 °C, with measurements of the diameter of the bacterial colony and clear zone (transparent agar surrounding colony) taken over 3 days. For supernatant PCL clearance, overnight cultures of bacteria were used to inoculate new cultures with a starting OD_600_ of 0.1, at OD_600_ 0.6 they were induced with 1 mM IPTG. After 22 hours growth, the cultures were centrifuged at 4000 × g for 20 min then the supernatant was filtered through 0.2 μm and 60 μL of filtered supernatant was spotted onto 1% PCL LBA plates and incubated at 37 °C. To assess thermostability of the enzymes, the filtered supernatant was concentrated ~ 2-3 × in an evaporating concentrator, heated at specific temperatures between 50-62.5 °C for 20 minutes then 60 μL spotted on 1% PCL LBA plates and incubated at 37 °C. The PCL clearance of the heat-treated supernatant was assessed over 4 days.

### PCL weight loss

For PCL weight loss assays, overnight cultures grown in LB broth at 37 °C were used to inoculate 10 mL fresh LB at OD_600_ 0.1 containing sterilised PCL beads. The cultures were grown at 37 °C with agitation until OD_600_ 0.6, then were induced with 1 mM IPTG and grown for 5 days. The culture media was refreshed after 3 days due to Ampicillin instability (culture collected, centrifuged at 5000 × g for 15 minutes, pellet resuspended in fresh media and returned to flask). The PCL beads were collected using a fine mesh sieve (63 μm), rinsed with distilled water, washed on a room temperature rocker in 2% sodium dodecyl sulphate overnight to remove biofilm, rinsed with distilled water and dried before weighing. Control weight loss flasks with uninoculated media and PCL were used to correct the weight loss measurements to account for instrument error or nonbiological degradation from spontaneous hydrolysis so that the control had 0% weight loss. Statistical analysis and graph construction was performed in Prism (version 9.4.1) throughout. Normality of data was assessed with a Shapiro-Wilk test in Prism. An alpha of 0.05 was used throughout, * is *p* < 0.05, ** is *p* < 0.01, *** is *p* < 0.001. Multiple comparisons were corrected with Dunnett’s test, and adjusted *p* value presented. F was calculated in Prism for each test.

### Biofilm assay

Overnight cultures grown in LB broth at 37 °C were used to inoculate 3 mL fresh LB at OD_600_ 0.1 in glass test tubes then grown with agitation at 37 °C. The cultures were induced with 0.5 mM IPTG at ~ OD_600_ 0.6 and grown for a total of 24 hours. The biofilm assay was performed with 5 mL serological pipettes to reduce biofilm dislodging. The culture was removed, then the test tube was washed 3 times with distilled water, 5 mL 0.1% crystal violet was added for 12 minutes then removed, and the test tube was washed 5 times with distilled water. The test tubes were fully air dried at room temperature then photographed.

### CFU analysis on PCL beads

Overnight cultures of bacteria were used to inoculate new cultures with a starting OD_600_ of 0.1, at OD_600_ 0.6 they were induced with 1 mM IPTG. After mixing the culture for 5 minutes with agitation, 150 μL of induced culture was added to a well of a 96-well plate containing a sterile PCL bead, in triplicate. After 24 hours, the PCL bead was removed with sterile tweezers and dip washed 3 times in sterile distilled water to remove unattached bacteria, then placed in 1 mL sterile phosphate buffered saline (PBS) in a 1.5 mL microcentrifuge tube. The tube was then sonicated for 10 minutes in a Camlab Transsonic T460 bath at 35 kHz to remove bacteria attached to the plastic surface. Serial dilutions were made from the PBS suspension and spotted in triplicate on LB agar containing appropriate antibiotics and grown for 16 hours at 37 °C to calculate CFU formed on the PCL beads for the different strains.

### Nanodrop absorbance assay for PET degradation

Adapting a previously published absorbance assay method to detect PET breakdown products, we used a nanodrop (Implen^TM^ NanoPhotometer ® NP80, path 0.67 mm) to measure PET-degrading activity of the novel enzymes^42^. Absorbance readings between 200 and 900 nm were taken to detect the presence of PET breakdown products TPA and MHET, which have an absorbance peak at 230-260 nm. TPA (Sigma-Aldrich) was dissolved in enzyme reaction buffer (50 mM glycine-NaOH pH 9, 50 mM NaCl, 10% (v/v) dimethyl sulfoxide) at different concentrations and absorbance measured on the nanodrop to confirm the expected peak (blanked against buffer not containing TPA). Overnight cultures of bacteria were used to inoculate new cultures with a starting OD_600_ of 0.1, at OD_600_ 0.6 they were induced with 1 mM IPTG. After 24 hours growth, the cultures were centrifuged at 4000 × g for 20 min then the supernatant was filtered through 0.2 μm. Supernatants were purified using Amicon® Ultra-15 Centrifugal Filter Units 3 kDa MWCO to concentrate the sample from 12 mL to ~ 1.2 mL to remove amino acids and small peptides present in the LB. The sample was then re-diluted in sterile distilled water to 12 mL and total protein concentration was normalised to 0.06 mg/ mL using a Bradford assay (Thermo Scientific^TM^ Pierce^TM^). 500 μL of filtered supernatant was then mixed with ~75 mg sterilised PET powder (Goodfellow <300 um, >40% crystallinity) in a 1.5 mL microcentrifuge tube. 500 μL was also added to microcentrifuge tubes not containing PET powder, as a control. The filtered supernatant also underwent acetone precipitation by mixing with 4 × volume cold acetone and incubating at -20 °C for 1 hour, briefly vortexing, centrifuging for 10 minutes at 15,000 × *g*, carefully removing the supernatant, and air-drying the precipitated protein pellet for 30 minutes. The protein pellet was resuspended in the enzyme reaction buffer and total protein concentration was normalised to 0.03 mg/ mL using a Bradford assay. 500 μL of precipitated protein was mixed with ~75 mg sterilised PET powder in a 1.5 mL microcentrifuge tube, as above, control microcentrifuge tubes without PET were included. After 5-days incubation at 37 °C (shaking), the microcentrifuge tubes were centrifuged, liquid sample collected, and absorbance measured on the nanodrop. Samples were blanked on the nanodrop to the respective sample control microcentrifuge tube without PET to remove any background peaks present from the culture supernatant. Control samples of uninoculated cultures (LB control) and cultures without potential PET-degrading enzyme present (empty vector control) were used to confirm no presence of peaks. In cases where absorbance was above the detection limits (above 40), appropriate dilutions were made in water for supernatant samples and in enzyme reaction buffer for precipitated samples.

### Reporting summary

Further information on research design is available in the Nature Research Reporting Summary linked to this article.

### Supplementary information

SUPPLEMENTAL MATERIAL

Reporting Summary

## Data Availability

The datasets used and/or analysed during the current study available from the corresponding author on reasonable request.

## References

[1] Geyer R, Jambeck JR, Law KL (2017). Production, use, and fate of all plastics ever made. Sci. Adv..

[2] Charles, D. & Kimman, L. Plastic Waste Makers Index 2023. (Minderoo Foundation, 2023).

[3] OECD. Global Plastics Outlook. (2022).

[4] Geyer, R. in *Plastic Waste and Recycling* (ed Trevor M. Letcher) 13-32 (Academic Press, 2020).

[5] de Stephanis R, Giménez J, Carpinelli E, Gutierrez-Exposito C, Cañadas A (2013). As main meal for sperm whales: Plastics debris. Mar. Pollut. Bull..

[6] Laist, D. W. in *Marine* Debri*s:* Sour*ces, Impacts, and Solutions* (eds James M. Coe & Donald B. Rogers) 99-139 (Springer New York, 1997).

[7] Lee H, Shim WJ, Kwon J-H (2014). Sorption capacity of plastic debris for hydrophobic organic chemicals. Sci. Total Environ..

[8] García-Gómez, J. C., Garrigós, M. & Garrigós, J. Plastic as a vector of dispersion for marine species with invasive potential. a review. *Front. Ecol. Evol.* **9**, 10.3389/fevo.2021.629756 (2021).

[9] Beloe CJ, Browne MA, Johnston EL (2022). Plastic debris as a vector for bacterial disease: an interdisciplinary systematic review. Environ. Sci. Technol..

[10] Lehel J, Murphy S (2021). Microplastics in the food chain: food safety and environmental aspects. Rev. Environ. Contam Toxicol..

[11] Duis K, Coors A (2016). Microplastics in the aquatic and terrestrial environment: sources (with a specific focus on personal care products), fate and effects. Environ. Sci. Eur..

[12] Groh KJ (2019). Overview of known plastic packaging-associated chemicals and their hazards. Sci. Total Environ..

[13] Jung J-W, Kang J-S, Choi J, Park J-W (2020). Chronic toxicity of endocrine disrupting chemicals used in plastic products in Korean resident species: Implications for aquatic ecological risk assessment. Ecotoxicol. Environ. Saf..

[14] Tetu SG (2019). Plastic leachates impair growth and oxygen production in Prochlorococcus, the ocean’s most abundant photosynthetic bacteria. Commun. Biol..

[15] Teuten EL (2009). Transport and release of chemicals from plastics to the environment and to wildlife. Philos. Trans. R. Soc. Lond. B Biol. Sci..

[16] Parvin F, Tareq SM (2021). Impact of landfill leachate contamination on surface and groundwater of Bangladesh: a systematic review and possible public health risks assessment. Appl. Water Sci..

[17] Omar H, Rohani S (2015). Treatment of landfill waste, leachate and landfill gas: A review. Front. Chem. Sci. Eng..

[18] Chianga PC, You JH, Chang S-C, Wei Y-H (1992). Identification of toxic PAH compounds in emitted particulates from incineration of urban solid wastes. J. Hazard. Mater..

[19] Li C-T, Zhuang H-K, Hsieh L-T, Lee W-J, Tsao M-C (2001). PAH emission from the incineration of three plastic wastes. Environ. Int..

[20] Hopewell J, Dvorak R, Kosior E (2009). Plastics recycling: challenges and opportunities. Philos. Trans. R. Soc. Lond. Ser. B, Biol. Sci..

[21] Shamsuyeva M, Endres H-J (2021). Plastics in the context of the circular economy and sustainable plastics recycling: Comprehensive review on research development, standardization and market. Compos. Part C: Open Access.

[22] Herrero Acero E (2011). Enzymatic surface hydrolysis of PET: effect of structural diversity on kinetic properties of cutinases from Thermobifida. Macromolecules.

[23] Ribitsch D (2012). Characterization of a new cutinase from Thermobifida alba for PET-surface hydrolysis. Biocat. Biotransf..

[24] Ribitsch, D. et al. A New Esterase from Thermobifida halotolerans Hydrolyses Polyethylene Terephthalate (PET) and Polylactic Acid (PLA). *Polymers* **4**, 10.3390/polym4010617 (2012).

[25] Yoshida S (2016). A bacterium that degrades and assimilates poly (ethylene terephthalate). Sci. (N. Y.).

[26] Danso D (2018). New insights into the function and global distribution of polyethylene terephthalate (PET)-degrading bacteria and enzymes in marine and terrestrial metagenomes. Appl. Environ. Microbiol..

[27] Almeida, E. L., Carrillo Rincón, A. F., Jackson, S. A. & Dobson, A. D. W. In silico Screening and Heterologous Expression of a Polyethylene Terephthalate Hydrolase (PETase)-Like Enzyme (SM14est) With Polycaprolactone (PCL)-Degrading Activity, From the Marine Sponge-Derived Strain Streptomyces sp. SM14. *Frontiers in Microbiology* **10**, 10.3389/fmicb.2019.02187 (2019). 10.3389/fmicb.2019.02187 PMC6779837 31632361

[28] Howard, S. A. et al. Enrichment of native plastic-associated biofilm communities to enhance polyester degrading activity. *Environ Microbiol*, 10.1111/1462-2920.16466 (2023). 10.1111/1462-2920.16466 PMC10947123 37515381

[29] Kenny ST (2008). Up-Cycling of PET (Polyethylene Terephthalate) to the Biodegradable Plastic PHA (Polyhydroxyalkanoate). Environ. Sci. Technol..

[30] Tournier V (2020). An engineered PET depolymerase to break down and recycle plastic bottles. Nature.

[31] Brott S (2022). Engineering and evaluation of thermostable IsPETase variants for PET degradation. Eng. Life Sci..

[32] Rennison, A., Winther, J. R. & Varrone, C. Rational Protein Engineering to Increase the Activity and Stability of IsPETase Using the PROSS Algorithm. *Polymers (Basel)* **13**, 10.3390/polym13223884 (2021). 10.3390/polym13223884 PMC8621346 34833182

[33] Meng X (2021). Protein engineering of stable IsPETase for PET plastic degradation by Premuse. Int. J. Biol. Macromolecules.

[34] Son HF (2019). Rational protein engineering of thermo-stable PETase from Ideonella sakaiensis for Highly Efficient PET Degradation. ACS Catal..

[35] Liu B (2018). Protein crystallography and site-direct mutagenesis analysis of the poly(ethylene terephthalate) hydrolase PETase from ideonella sakaiensis. ChemBioChem.

[36] Ma Y (2018). Enhanced Poly(ethylene terephthalate) hydrolase activity by protein engineering. Engineering.

[37] Lu H (2022). Machine learning-aided engineering of hydrolases for PET depolymerization. Nature.

[38] Donlan RM (2002). Biofilms: microbial life on surfaces. Emerg. Infect. Dis..

[39] Valentini M, Filloux A (2016). Biofilms and Cyclic di-GMP (c-di-GMP) Signaling: Lessons from Pseudomonas aeruginosa and Other Bacteria. J. Biol. Chem..

[40] Joo S (2018). Structural insight into molecular mechanism of poly(ethylene terephthalate) degradation. Nat. Commun..

[41] Gräslund S (2008). Protein production and purification. Nat. Methods.

[42] Zhong-Johnson EZL, Voigt CA, Sinskey AJ (2021). An absorbance method for analysis of enzymatic degradation kinetics of poly(ethylene terephthalate) films. Sci. Rep..

[43] Austin HP (2018). Characterization and engineering of a plastic-degrading aromatic polyesterase. Proc. Natl. Acad. Sci..

[44] McCarthy RR (2017). Cyclic-di-GMP regulates lipopolysaccharide modification and contributes to Pseudomonas aeruginosa immune evasion. Nat. Microbiol..

[45] Leech, J. T. *Development of an Escherichia coli Biofilm Platform for use in Biocatalysis*, University of Birmingham, (2017).

[46] Chen S (2008). Identification and characterization of bacterial cutinase. J. Biol. Chem..

[47] Basili, M. et al. Major Role of Surrounding Environment in Shaping Biofilm Community Composition on Marine Plastic Debris. *Frontiers in Marine Science* **7**, 10.3389/fmars.2020.00262 (2020).

[48] Lobelle D, Cunliffe M (2011). Early microbial biofilm formation on marine plastic debris. Mar. Pollut. Bull..

[49] Morohoshi T (2018). Biofilm formation and degradation of commercially available biodegradable plastic films by bacterial consortiums in freshwater environments. Microbes Environ..

[50] Gilan I, Hadar Y, Sivan A (2004). Colonization, biofilm formation and biodegradation of polyethylene by a strain of Rhodococcus ruber. Appl. Microbiol. Biotechnol..

[51] Mor R, Sivan A (2008). Biofilm formation and partial biodegradation of polystyrene by the actinomycete Rhodococcus ruber. Biodegradation.

[52] Kelley LA, Mezulis S, Yates CM, Wass MN, Sternberg MJE (2015). The Phyre2 web portal for protein modeling, prediction and analysis. Nat. Protoc..

