# Supplementary material for: Modulating biofilm can potentiate activity of novel plastic-degrading enzymes
Source: NPJ Biofilms Microbiomes. 2023 Oct 3;9:72. doi: 10.1038/s41522-023-00440-1 (PMC10547765; doi:10.1038/s41522-023-00440-1)
Supplement: Supplementary file 1 — SUPPLEMENTAL MATERIAL [file 41522_2023_440_MOESM1_ESM.pdf]

Supplementary file

Supplementary Table 1. Known polyester-degrading enzymes used for genome mining

Enzyme name	Accession
HiC	QAY29138
FsC	AAA33335
TfCut2	CBY05530
Tha_Cut1	ADV92525
Thh_Est	AFA45122
Thc_Cut1	ADV92526
Thc_Cut2	ADV92527
Thf42_Cut1	ADV92528
IsPETase	GAP38373
Tcur1278	ACY96861
Tcur0390	ACY95991
LCC	AEV21261
PET12	AKJ29164
TfH	AAZ54921
Cut190	BAO42836
PET2	ACC95208
PET5	CCK74972
PE-H	OWL88088
TfCut1	CBY05529
cut_1	AET05798
BsEstB	ADH43200

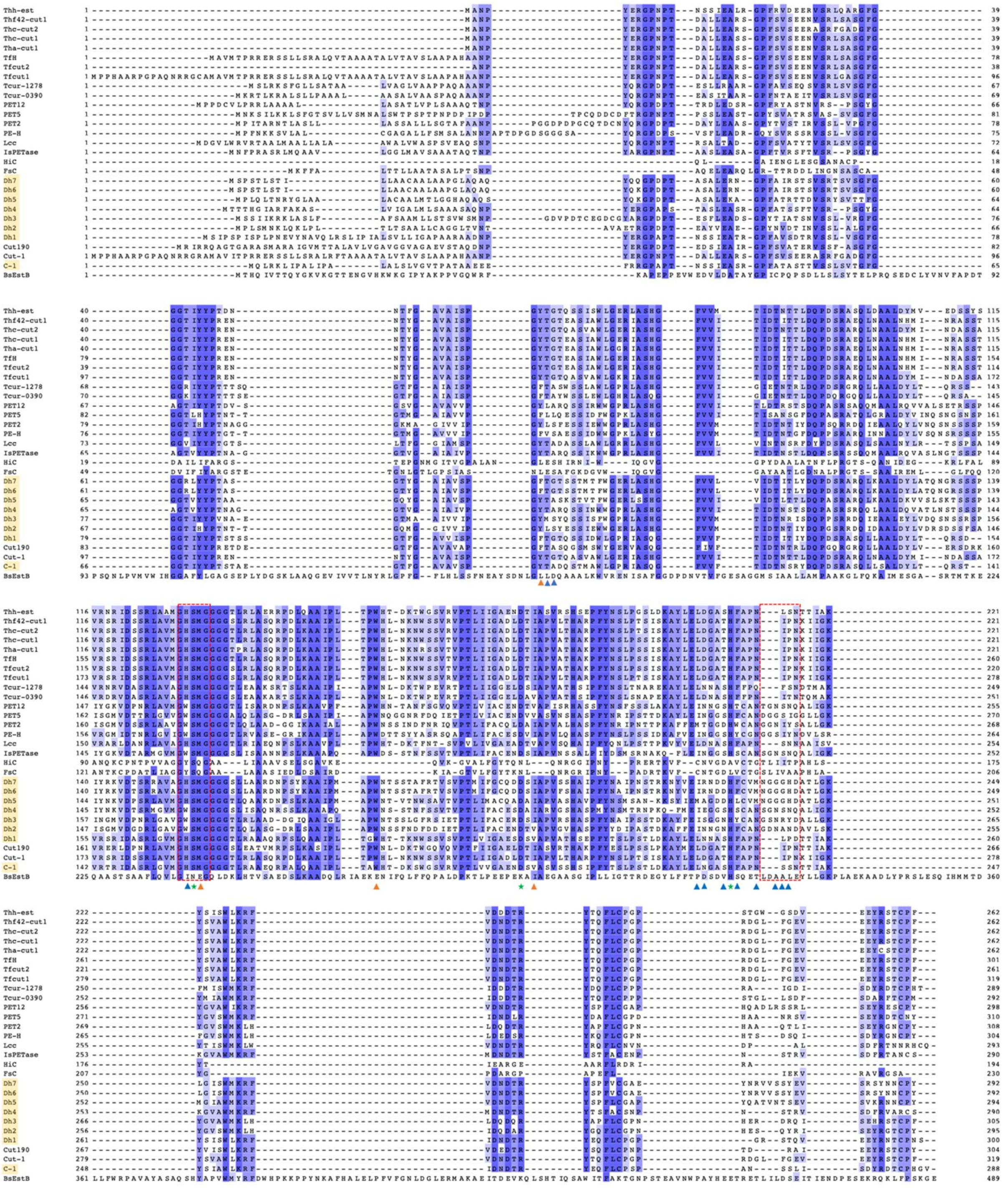

Supplementary Figure 1. Protein sequence alignment of polyester-degrading enzymes. Protein sequences of 21 known polyester-degrading enzymes and 8 novel potential polyester-degrading enzymes from this study (highlighted in yellow). Aligned with MUSCLE and conservation coloured by percent identity in Jalview. Annotation – 1st red box is Gly–x1–Ser–x2–Gly motif, 2nd red box is extended loop region, orange triangles demarcate subsite 1, blue triangles demarcate subsite 2 and green stars demarcate the catalytic triad.

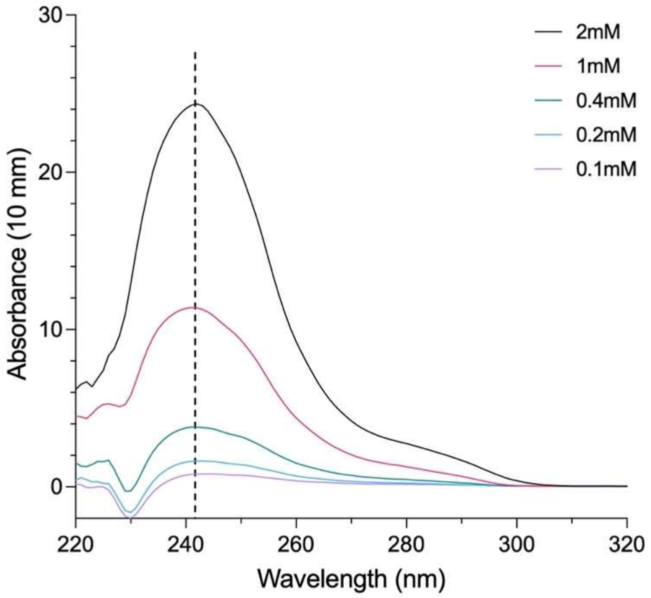

Supplementary Figure 2. TPA absorbance spectra. TPA was dissolved in enzyme reaction buffer at different concentrations and nanodrop absorbance readings were taken, mean curve of triplicate readings.

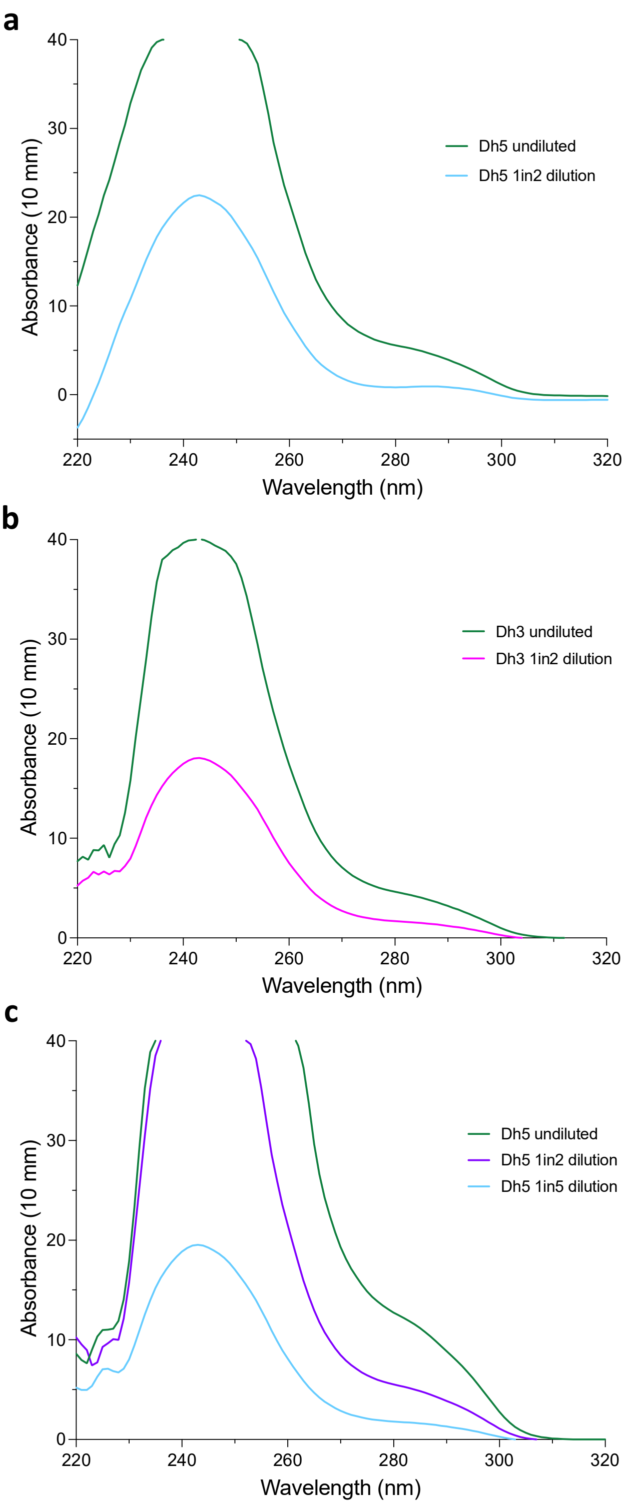

Supplementary Figure 3. Original undiluted absorbance readings of PET degradation products. Undiluted and 1 in 2 or 1 in 5 dilution spectra for (a) Dh5 supernatant samples, (b) Dh3 precipitated samples and (c) Dh5 precipitated samples. Corresponding to spectra in Fig. 4, mean curve of 4 biological replicates from the same day. The nanodrop limit of detection is 40, so values of 40 are excluded from the graph.

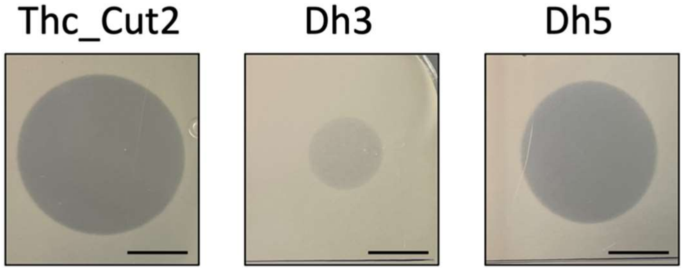

Supplementary Figure 4. Confirmation of enzyme activity after acetone precipitation. Filtered supernatant of induced cultures underwent acetone precipitation, after dissolving the protein pellet in enzyme reaction buffer, 20 μl was spotted onto 1% PCL LBA and incubated at 37°C and photographed. Representative image of 3 biological repeats. Scale bar 1 cm.

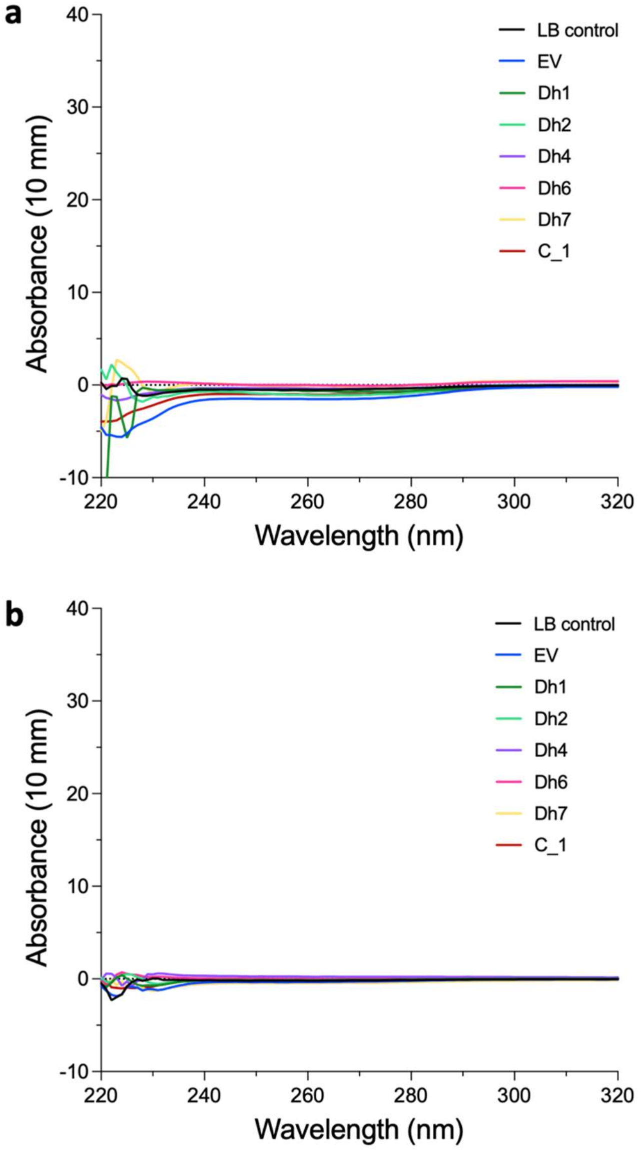

Supplementary Figure 5. Absorbance spectra for novel enzymes. (a) Supernatant and (b) precipitated protein samples containing Dh1, Dh2, Dh4, Dh6, Dh7 and C_1 were mixed with PET powder for 5 days and read on a nanodrop. Mean curve of 3 (supernatant) and 2 (precipitated proteins) biological replicates from the same day.

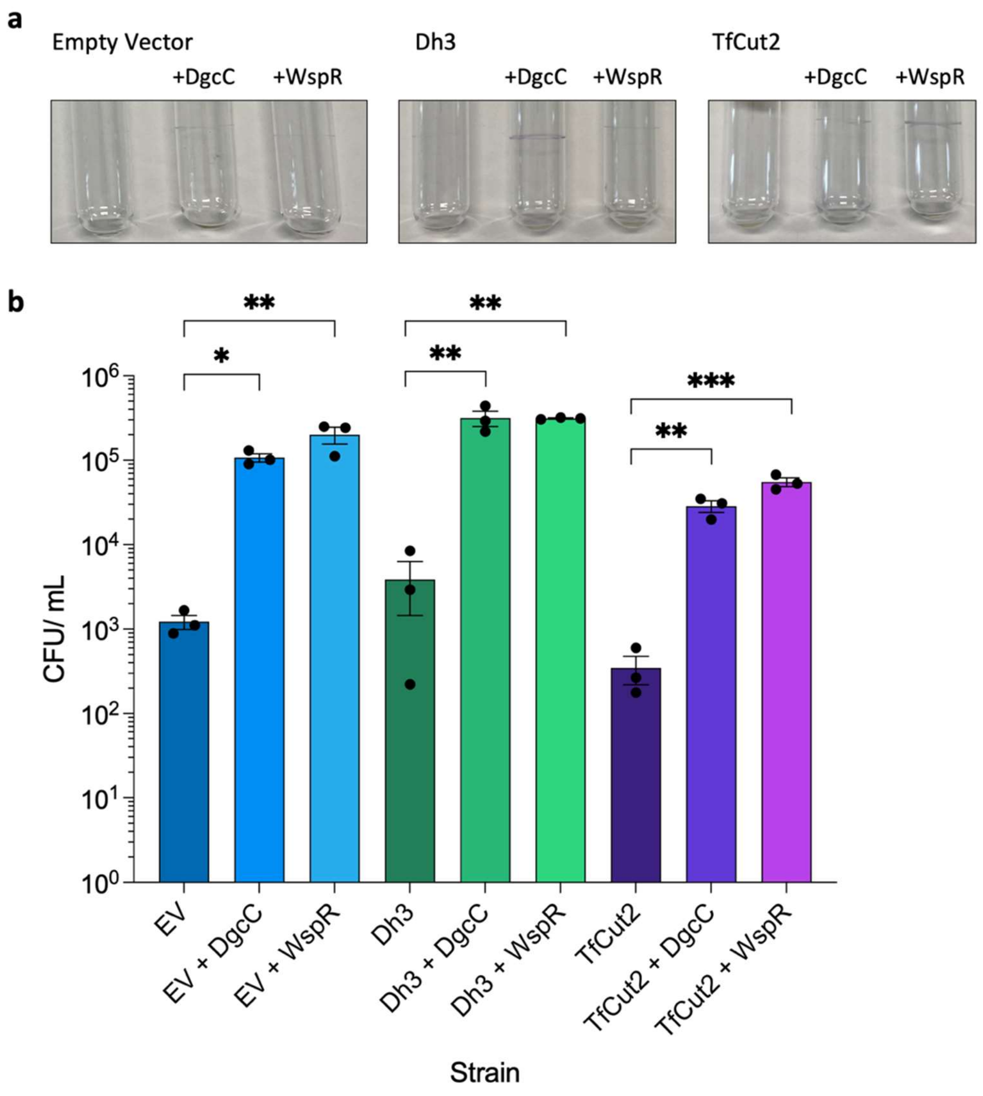

Supplementary Figure 6. Biofilm analysis of co-expression of DGC's with PET-degrading enzymes. (a) Biofilm assay of strains with and without co-expression of DgcC and WspR. Cultures were grown in test tubes for 24 hours, tubes were rinsed and stained with crystal violet, rinsed and dried then imaged. Each image is one enzyme (first image is BL21 (DE3) empty vector control), first tube is not expressed with DGC, second tube is expressed with DgcC, third tube is expressed with WspR. Representative image of 5 biological repeats. (b) CFU from PCL bead surface of empty vector, Dh3 and TfCut2 with and without co-expression of DgcC or WspR. Mean and SEM of three biological repeats (each consisting of the mean of three technical replicates). Ordinary one-way ANOVA with multiple comparisons was used to compare whether addition of DGC significantly changed CFU/ mL to the strain without. *p*-values are 0.0314 for EV vs EV + DgcC and 0.0038 for EV vs EV + WspR, F 13.85. *p*-values 0.0022 for Dh3 vs Dh3 + DgcC and 0.0022 for Dh3 vs Dh3 + WspR, F 22.37. *p*-values 0.0088 for TfCut2 vs TfCut2 + DgcC, 0.0003 for TfCut2 vs TfCut2 + WspR, F 35.53.
